# Yohimbine Promotes Cardiac NE Release and Prevents LPS-Induced Cardiac Dysfunction via Blockade of Presynaptic α_2A_-Adrenergic Receptor

**DOI:** 10.1371/journal.pone.0063622

**Published:** 2013-05-14

**Authors:** Yiyang Wang, Xiaohui Yu, Faqiang Wang, Yuan Wang, Yanping Wang, Hongmei Li, Xiuxiu Lv, Daxiang Lu, Huadong Wang

**Affiliations:** 1 Department of Pathophysiology, School of Medicine, Jinan University, Guangzhou, Guangdong, China; 2 Key Laboratory of State Administration of Traditional Chinese Medicine of the People’s Republic of China, School of Medicine, Jinan University, Guangzhou, Guangdong, China; Scuola Superiore Sant’Anna, Italy

## Abstract

Myocardial depression is an important contributor to mortality in sepsis. We have recently demonstrated that α_2_-adrenoceptor (AR) antagonist, yohimbine (YHB), attenuates lipopolysaccharide (LPS)-induced myocardial depression. However, the mechanisms for this action of YHB are unclear. Here, we demonstrated that YHB decreased nitric oxide (NO) and tumor necrosis factor-alpha (TNF-α) levels in the myocardium and plasma, attenuated cardiac and hepatic dysfunction, but not kidney and lung injuries in endotoxemic mice. Immunohistochemical analysis revealed that cardiac α_2A_-AR was mostly located in sympathetic nerve presynaptic membrane; YHB decreased cardiac α_2A_-AR level and promoted cardiac norepinephrine (NE) release in endotoxemic mice. Reserpine that exhausted cardiac NE without markedly decreasing plasma NE level abrogated the inhibitory effects of YHB on cardiac TNF-α and iNOS expression as well as cardiac dysfunction, but not the suppressive effects of YHB on plasma TNF-α and NO elevation in LPS-challenged mice. Furthermore, both reserpine and YHB significantly inhibited LPS-induced myocardial apoptosis. α_1_-AR, β_2_-AR, but not β_1_-AR antagonists reversed the inhibitory effect of YHB on LPS-stimulated myocardial apoptosis. However, β_1_-AR antagonist attenuated LPS-caused cardiomyocyte apoptosis, partly abolished the protective effect of YHB on the left ventricular ejection fraction in endotoxemic mice. Altogether, these findings indicate that YHB attenuates LPS-induced cardiac dysfunction, at least in part, through blocking presynaptic α_2A_-AR and thus increasing cardiac NE release. YHB-elevated cardiac NE improves cardiac function via suppressing cardiac iNOS and TNF-α expression, activating β_1_-AR and inhibiting cardiomyocyte apoptosis through α_1_- and β_2_-AR in endotoxemic mice. However, cardiac β_1_-AR activation promotes LPS-induced cardiomyocyte apoptosis.

## Introduction

Severe sepsis remains a leading cause of death in the intensive care unit [1]. Myocardial dysfunction is a key contributor to mortality in septic patients [2]. Therefore, it is very important to develop new therapeutic approaches to septic cardiac dysfunction.

Lipopolysaccharide (LPS), which is found to play an important role in the pathogenesis of sepsis, can cause cardiac dysfunction manifested as depression of left ventricular ejection fraction (EF) and decreased cardiac output (CO) [3]. Although increasing evidence showed that myocardial apoptosis and production of inflammatory mediators, including tumor necrosis factor-α (TNF-α) and nitric oxide (NO), contributed to LPS-induced cardiac dysfunction [4]–[6], the mechanisms for this myocardial dysfunction are far from clear and no specific drugs can improve septic cardiac dysfunction in clinical practice [7]. Recently, we demonstrated that pretreatment with yohimbine (YHB), an α_2_-adrenoceptor (AR) antagonist, significantly protected against myocardial dysfunction in endotoxemic mice [8], suggesting that α_2_-AR activation is an important contributor to LPS-caused cardiac dysfunction. However, the precise mechanisms responsible for this action of YHB remain undefined.

To date, three α_2_-AR subtypes (α_2A_, α_2B_, α_2C_) have been identified, which differ in their pharmacological properties and tissue distribution [9]. Wang, et al. have demonstrated that norepinephrine (NE) promotes LPS-induced TNF-α production in the Kupffer cells via stimulating α_2A_ –AR [10], they also found that blockade of α_2A_ -AR inhibited inflammatory responses and improve survival in septic rats [11]. Furthermore, α_2A_-AR is found in other macrophages [12], LPS-activated macrophages adhered to cardiomyocytes decrease myocardial contractile function via TNF-α and NO [13]. Hence, one possibility is that the protection of YHB against LPS-triggered myocardial dysfunction may be related to the inhibition of α_2A_-AR in macrophages. However, inhibition of α_2_-AR by YHB can reduce cardiac injury, but not pulmonary injury in LPS-challenged animals [8], [14], indicating that YHB may have an organ-specific protective action in endotoxemia. It has demonstrated that YHB can increase NE release via blocking presynaptic α_2_-AR in myocardium [15] and β-AR activation inhibits LPS-induced myocardial production of TNF-α [16]. Therefore, besides α_2_-AR in macrophages, cardiac presynaptic α_2_-AR may be involved in the protection of YHB against LPS-induced cardiac dysfunction. To test this hypothesis, we investigated the roles of cardiac presynaptic α_2_-AR and NE in YHB-induced protection against LPS-induced cardiac dysfunction in the current study.

## Methods

### Animals and Animal Procedures

Male BALB/c mice (22–24 g, 7–9 weeks old) were obtained from the medical laboratory animal center of Guangdong province (Guangzhou, China). All experiments were performed in compliance with the Guide for the Care and Use of Laboratory Animals published by the US National Institutes of Health, and approved by the Animal Care and Use Committee at School of Medicine, Jinan University. The mice were anesthetized with isoflurane or pentobarbital (100 mg/kg) under necessary conditions. The adequacy of the anesthesia was monitored by failure to respond to a skin incision, disappearance of the corneal reflex and loss of the pedal reflex, and every effort was made to minimize suffering. LPS, YHB, prazosin, atenolol, ICI118551 and reserpine were purchased from Sigma Aldrich (St. Louis, Mo, USA). In the separate experiment, YHB (0.5, 1, 2 or 4 mg/kg) was administered intragastrically; LPS (20 mg/kg) or normal saline was injected intraperitoneally 1 h after treatment with YHB or water; prazosin (2 mg/kg), atenolol (10 mg/kg), ICI118551 (10 mg/kg) or vehicle was administered intraperitoneally and followed immediately by YHB or water treatment; four days before YHB or water treatment, reserpine (RSP, 4.5 mg/kg) was given subcutaneously once a day for 2 consecutive days.

### Echocardiography

Echocardiography was performed under isoflurane anesthesia (2%) using the Vevo770™ high resolution imaging system (Inc., Toronto, Ontario, Canada) at 12 h post LPS or normal saline injection. Two-dimensional M-mode imaging from parasternal short-axis view at the level of the papillary muscles and the apical four-chamber view were obtained, and ascending aortic flow velocity was recorded using the continuous Doppler wave mode as described previously [8]. EF, left ventricular end-diastolic volume (LVEDV), left ventricular end-systolic volume (LVESV), stroke volume (SV) and CO were calculated by the software of Vevo770™ imaging system. The echocardiography measurements were interpreted by the investigator blinded to treatment, and the data were averaged from at least three consecutive cardiac cycles.

### Assessment of Lung Wet-to-dry Weight Ratio, Hepatic and Renal Function

The pulmonary edema was assessed by measurement of lung wet-to-dry (W/D) weight ratio as described previously [14]. Serum alanine aminotransferase (ALT) and blood urea nitrogen (BUN) were examined using automatic biochemical analyzer (7600-020, HITA CHI Inc., Tokyo, Japan).

### Detection of Nitric Oxide and Tumor Necrosis Factor-α

TNF-α concentrations were measured by enzyme-linked immunosorbent assay (ELISA) according to the manufacturer’s protocol (R&D Systems, Inc, Minneapolis, Minn). NO was measured as its stable oxidative metabolites, nitrite, using Greiss reagent system (Promega, Madison, Wis).

### Western Blot Analysis

Equal amounts of protein from cardiac and lung tissues were subjected to separation on 10% SDS- polyacrylamide gel electrophoresis, and then electrotransfered to nitrocellulose membranes. Following blockage of nonspecific binding sites for 1 h, the membranes were incubated overnight at 4°C in 1∶1000 dilution of antibodies against glyceraldehyde-3-phosphate dehydrogenase (GAPDH, Cell Signaling Technology, MA, USA), iNOS (Cell Signaling Technology, MA, USA), α_2A_-AR, α_2C_-AR (Abcam, Cambridge, UK) or 1∶500 dilution of antibodies against α_2B_-AR (Santa Cruz, CA, USA), respectively. Then, the membranes were incubated in a 1∶4000 dilution of horseradish peroxidase-conjugated secondary antibody for 1 h, subsequently developed with the enhanced chemiluminescence detection reagent. The results were quantified by scanning densitometry.

### Immunofluorescence Staining

The normal mouse hearts were harvested and 4% paraformaldehyde fixed. Myocardium sections were blocked in PBS with 5% BSA, following incubated with 1∶50 dilution of antibody against α_2A_-AR, α_2B_-AR or α_2C_-AR and synaptophysin (Santa Cruz, CA, USA) or CD34 (Abcam, Cambridge, UK) at 4°C overnight, respectively. Then, the sections were incubated with 1∶100 dilution of antibody against cardiac troponin I (Abcam, Cambridge, UK) at 4°C overnight. Afterwards, the sections were incubated with 1∶1000 dilution of secondary antibodies conjugated with Alexa Fluor® dyes (Invitrogen, Carlsbad, Calif, USA) for 1 h, and observed with laser confocal microscopy.

### Measurement of Norepinephrine

Cardiac and plasma NE concentrations were measured by ELISA (Alpco, Salem, NH, USA) according to manufacturer’s instructions.

### Determination of Cardiac Caspase-3/7 Activity

The activity of myocardial caspase-3/7 was determined by using the Apo-ONE^®^ homogeneous caspase-3/7 assay kit (Promega, Madison, Wisconsin, USA) according to the instruction of the manufacturer.

### Terminal Deoxynucleotidyl Transferase-mediated dUTP Nick-end-labeling Assay

Myocardial apoptosis was assessed by terminal deoxynucleotidyl transferase-mediated dUTP nick-end-labeling (TUNEL) assay using in situ apoptosis detection kit (Roche, Calif, USA). Myocardial sections were incubated with 1∶50 dilution of antibody against cardiac troponin I at 4°C overnight, then with TUNEL reaction mixture at 37°C for 60 min in the dark. The sections were rinsed with PBS, incubated with 1∶1000 dilution of secondary antibody conjugated with Alexa Fluor® dyes for 1 h. Finally, the sections were incubated with 1∶400 dilution of 4′,6-diamidino-2-phenylindole (DAPI) for 15 min, and observed with laser confocal microscopy. Apoptotic cells were counted in five randomly selected fields per section, the apoptotic index (AI) was calculated as follows: AI =  (number of TUNEL-positive cardiomyocytes/total number of cardiomyocytes) ×100.

### Statistic Analysis

All data were expressed as mean ± standard error of the mean (SEM). Significance of differences was analyzed with one-way ANOVA followed by Bonferroni post hoc analysis. *P*<0.05 were considered to be statistically significant.

## Results

### Yohimbine Attenuated Lipopolysaccharide-induced Cardiac and Hepatic Dysfunction, but not Kidney and Lung Injury

**Figure 1:**
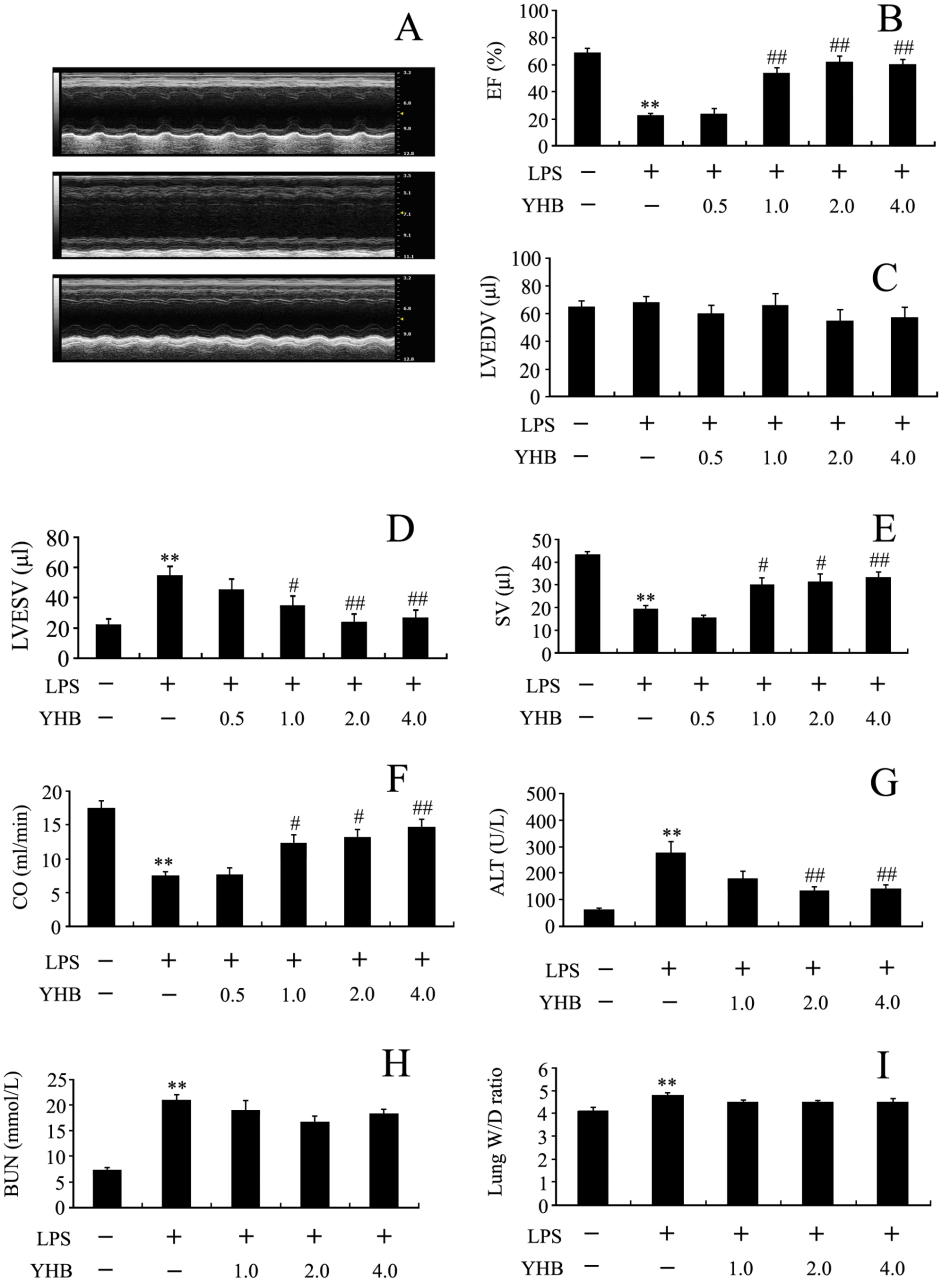
Effects of YHB (0.5, 1, 2 and 4 mg/kg) on organ injury in mice 12 h after LPS (20 mg/kg) injection. (A) Representative M-mode echocardiograms in control (upper), LPS (middle) and 1 mg/kg YHB+LPS (lower) groups. (B, C, D, E, F) Changes in ejection fraction (EF), left ventricular end-diastolic volume (LVEDV), left ventricular end-systolic volume (LVESV), stroke volume (SV) and cardiac output (CO). (G) Serum alanine aminotransferase (ALT) activity. (H) Blood urea nitrogen (BUN) level. (I) Lung wet-to-dry weight (W/D) ratio. *n* = 8–10. **P<0.01 compared with control group; ^#^ *P*<0.05, ^##^ *P*<0.01 compared with LPS group.

As shown in *Figure 1* *A–F*, EF, LVEDV, LVESV, CO and SV were calculated from echocardiography at 12 h after LPS injection. LPS or/and YHB did not markedly affect LVEDV in mice. However, LPS induced left ventricular contractile dysfunction in mice as evidenced by decreases in EF, CO and SV as well as an increase in LVESV compared with those in control. In contrast, YHB at a dose of 1, 2 or 4 mg/kg significantly reversed the LPS-induced changes in EF, LVESV, CO and SV. There was no significant difference in heart rate during echocardiographic measurements under anesthesia in various groups.

In addition, mice displayed markedly elevated W/D ratio, serum ALT activity and BUN level at 12 h after LPS challenge. YHB (2 and 4 mg/kg) significantly decreased serum ALT activity, but not lung W/D ratio and BUN level in LPS-challenged mice (*Figure 1* *G–I*).

### Yohimbine Inhibited Lipopolysaccharide-induced Tumor Necrosis Factor-α and Nitric Oxide Production in the Heart and Plasma

**Figure 2:**
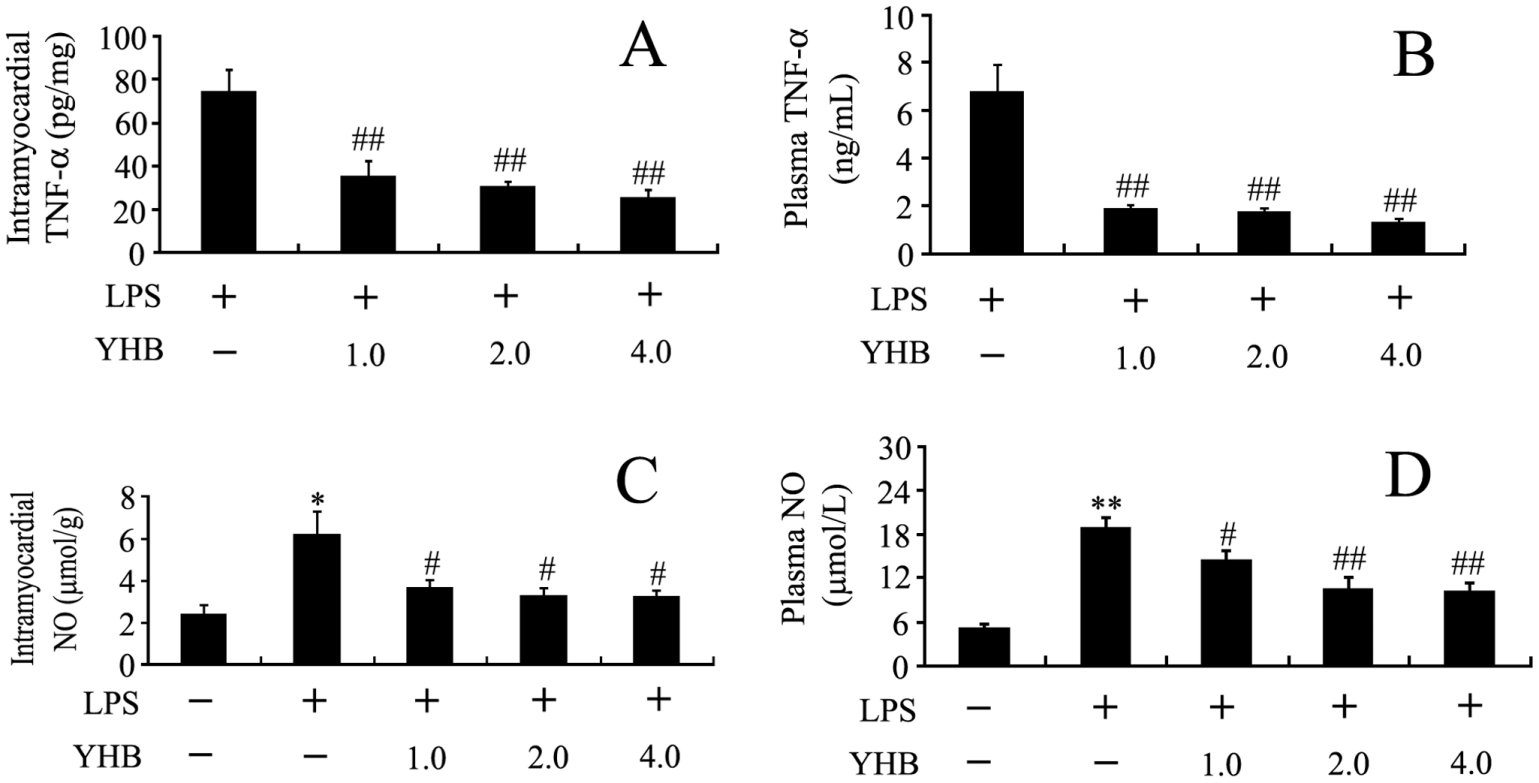
Effects of YHB (1, 2 or 4 mg/kg) on cardiac and plasma TNF-α and NO levels in LPS-challenged mice. (A and B) Cardiac and plasma TNF-α levels were examined at 1 h after 20 mg/kg LPS challenge (*n* = 10). (C and D) Cardiac and plasma NO levels were determined at 12 h after 20 mg/kg LPS injection (*n* = 10). *P<0.05, **P<0.01 compared with control group; ^#^ *P*<0.05, ^##^ *P*<0.01 compared with LPS group.

We further observed the effects of YHB on cardiac and plasma TNF-α and NO contents in endotoxemic mice. Cardiac and plasma TNF-α was not detectable in control mice. As shown in *Figure 2* *A and B,* the levels of TNF-α in the heart and plasma increased at 1 h after LPS treatment. YHB (1, 2 or 4 mg/kg) significantly reduced cardiac and plasma TNF-α levels at 1 h after LPS injection. The concentrations of cardiac TNF-α at 2 h after LPS injection were lower in 1 mg/kg YHB+LPS group (27.1±1.3 pg/mg) than LPS group (69.3±7.8 pg/mg, *n* = 10, *P*<0.01). Plasma TNF-α levels at 2 h after LPS injection were also lower in 1 mg/kg YHB+LPS group (1.91±0.28 ng/mL) than LPS group (9.08±0.80 ng/mL, *n* = 10, *P*<0.01). LPS significantly increased plasma and cardiac NO production at 12 h after LPS administration, which was inhibited by 1, 2 or 4 mg/kg YHB (*Figure 2* *C and D*).

### Yohimbine Reduced Cardiac α_2A_-AR Level,but not Lung α_2A_-AR Level in Lipopolysaccharide-treated Mice

**Figure 3:**
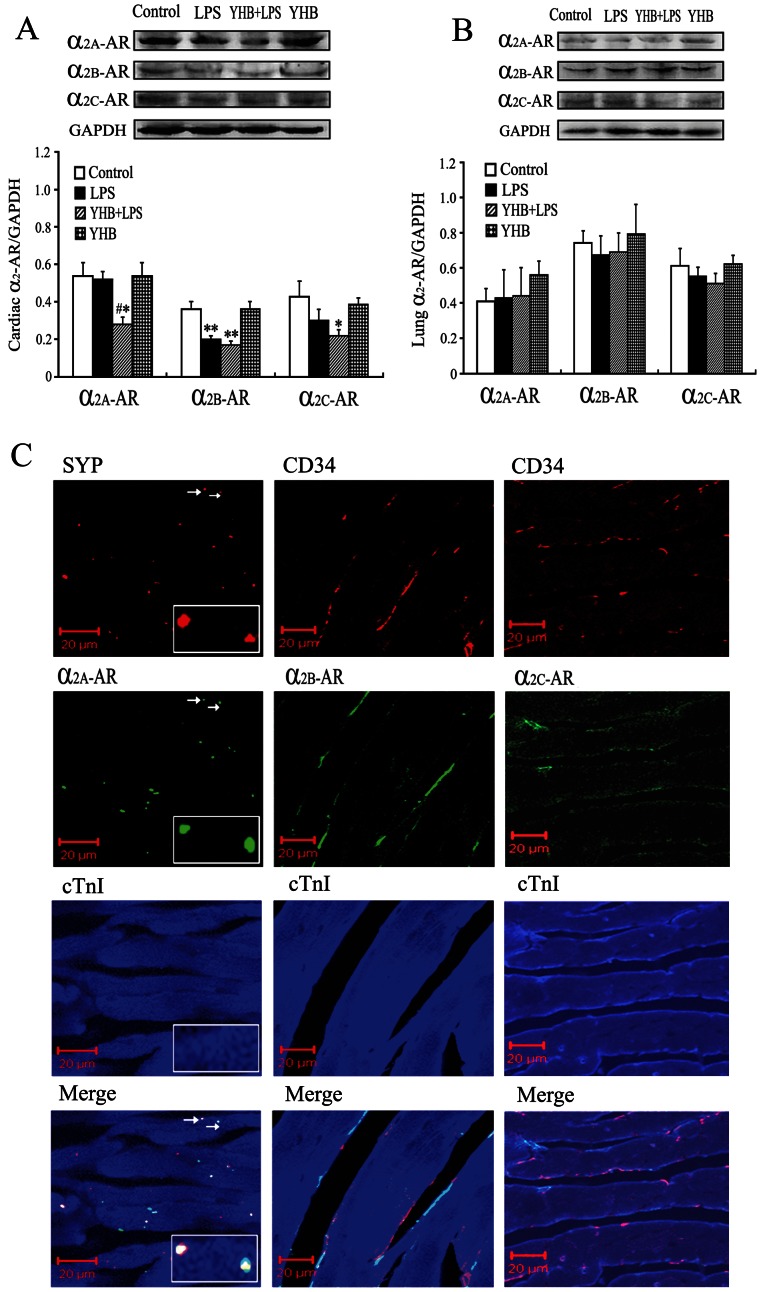
Cardiac and lung α_2_- AR levels and cardiac localization of α_2_- AR subtypes in YHB or/and LPS-challenged mice. (A and B) Levels of α_2A_, α_2B_ and α_2C_-AR protein in the heart and lung (*n* = 8). LPS (20 mg/kg) or normal saline was injected intraperitoneally 1 h after intragastrical treatment with YHB (1 mg/kg) or water, the α_2A_, α_2B_ and α_2C_-AR proteins were determined using Western blotting at 4 h after LPS injection. *P<0.05, **P<0.01 compared with control group; ^#^ *P*<0.05 compared with LPS group. (C) Representative confocal images of normal mouse cardiac sections. The sections were stained with antibodies against cardiac troponin I (blue), α_2_-AR subtypes (green) and synaptophysin (SYP, a mark for presynaptic terminals, red) or CD34 (a marker for endothelial cells, red). Insets, high-power magnification of the area indicated by arrows. Scale bar = 20 µm.

The levels of α_2A_, α_2B_ and α_2C_-AR in the heart and lung were determined using Western blotting. As shown in *Figure 3A and B*, LPS treatment for 4 h markedly decreased cardiac α_2B_-AR, but not α_2A_ and α_2C_-AR levels in mice. YHB significantly decreased α_2A_-AR level in the heart at 4 h after LPS treatment compared with LPS group. The cardiac α_2A_, α_2B_ and α_2C_-AR levels were lower in YHB+LPS group than control. In contrast, YHB did not significantly reduce α_2B_ and α_2C_-AR levels in the heart compared with LPS group, the levels of α_2_-AR subtypes in the lung at 4 h after LPS challenge were not markedly different among various groups.

### α_2A_-AR Predominantly Localized at Presynaptic Terminals in the Mouse Heart

The cardiac distribution of α_2_-AR subtypes was examined by combining immunofluorescence labeling for α_2A_-AR, α_2B_-AR or α_2C_-AR (green) with for synaptophysin (SYP, a mark for presynaptic terminals, red) or CD34 (a marker for endothelial cells, red). Dual-labeling myocardial sections were then stained with antibody against cardiac troponin I (blue). As shown in *Figure 3C*, α_2A_-AR was colocalized with SYP in the heart, presynaptic terminals labeled with both α_2A_-AR and SYP appear white, indicating that cardiac α_2A_-AR predominantly localized at presynaptic terminals. α_2B_-AR or α_2C_-AR (green fluorescence) was restricted to blood vessel wall, and observed as a linear pattern along the blood vessels. However, α_2B_-AR or α_2C_-AR staining was clearly apart from endothelial cell marker, CD34 (*Figure 3* *C*).

### Yohimbine Increased Cardiac and Plasma Norepinephrine Levels in Lipopolysaccharide-treated Mice

**Figure 4:**
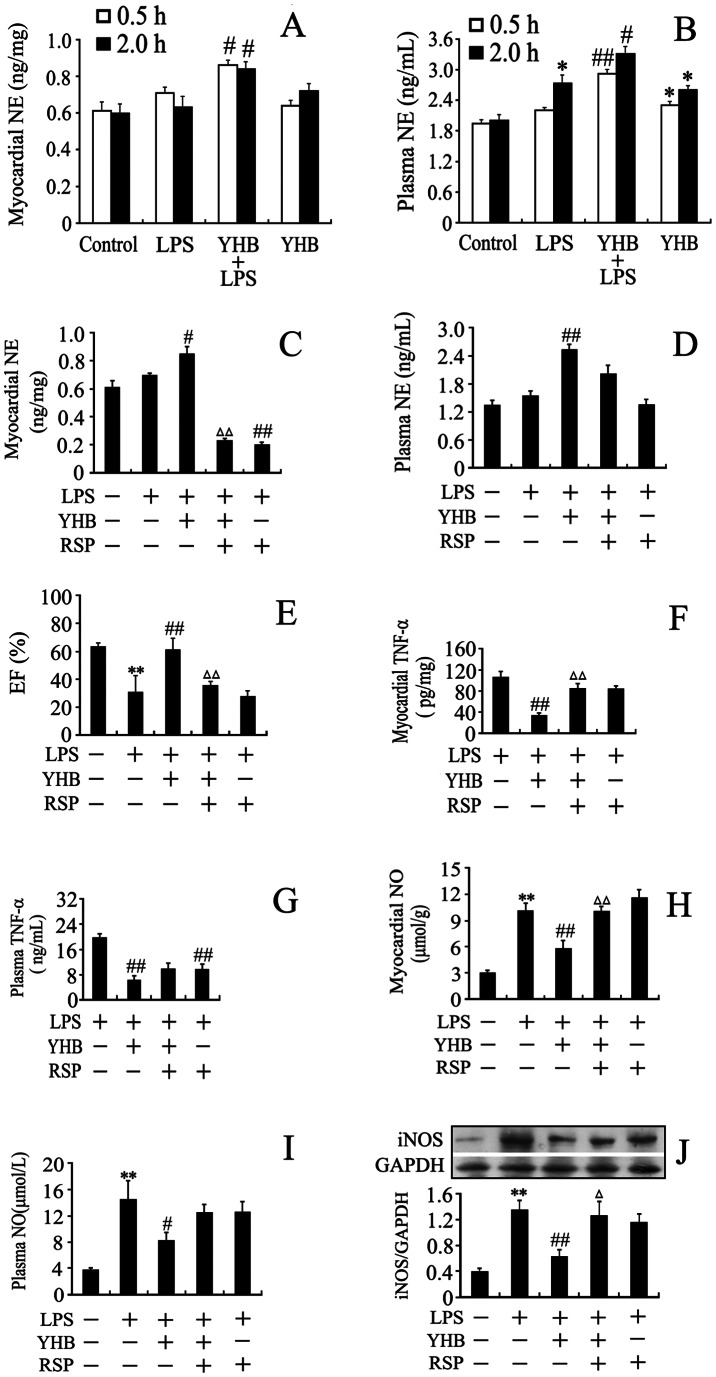
Effects of YHB or/and reserpine (RSP) on cardiac and plasma NE levels, TNF-α and NO production, myocardial inducible nitric oxide synthase (iNOS) expression and left ventricular EF in LPS-challenged mice. (A and B) Cardiac and plasma NE levels at 0.5 and 2 h after LPS or normal saline injection (n = 10). Mice were injected intraperitoneally with LPS (20 mg/kg) or normal saline at 1 h after intragastrical treatment with YHB (1 mg/kg) or water. In separate experiments, mice first received subcutaneous injection of RSP (4.5 mg/kg) or normal saline once a day for 2 consecutive days, then exposed to YHB (1 mg/kg) or/and LPS (20 mg/kg) on the 4th day after last RSP administration. LPS or normal saline was injected intraperitoneally at 1 h after YHB treatment. NE concentrations of the heart (C) and plasma (D) was detected at 0.5 h after LPS injection (*n* = 8). (E) Left ventricular EF 12 h after LPS injection (*n* = 8). (F and G) TNF-α production of the heart and plasma at 2 h after LPS challenge (*n* = 10). (H and I) Cardiac and plasma NO levels at 12 h after LPS injection (*n* = 8). (J) Cardiac iNOS expression was detected by Western blot assay at 6 h after LPS challenge (*n* = 7). *P<0.05, **P<0.01 compared with control group; ^#^ *P*<0.05, ^##^ *P*<0.01 compared with LPS group; ^Δ^ *P*<0.05, ^ΔΔ^ *P*<0.01 compared with YHB+LPS group.

Since α_2A_-AR subtype contributes to presynaptic feedback inhibition of NE release [9], we further investigated the changes in cardiac and plasma NE concentrations. As shown in *Figure 4A and B*, LPS increased NE concentration in plasma 2 h after LPS injection, but not in the heart. The cardiac and plasma levels of NE at 0.5 and 2 h after LPS injection were higher in YHB+LPS group than those in LPS group. Plasma NE concentration was elevated in YHB group compared with control.

Reserpine exhausted cardiac norepinephrine and eliminated the inhibitory effects of yohimbine on cardiac dysfunction, tumor necrosis factor-α and nitric oxide production as well as inducible nitric oxide synthase (iNOS) expression in lipopolysaccharide-challenged mice.

To test whether YHB improved myocardial dysfunction in LPS-treated mice through increased cardiac NE release, we used reserpine (RSP) to exhaust cardiac NE in LPS-treated mice. It was demonstrated that there was no NE recovery in heart from the 4th day to 10th day after subcutaneous RSP treatment [17]. In the present study, mice first received subcutaneous injections of RSP (4.5 mg/kg) once a day for 2 consecutive days, then exposed to YHB or/and LPS on the 4th day after last administration of RSP. At 0.5 h after LPS challenge, the cardiac and plasma levels of NE in YHB+LPS group were significantly higher than those in LPS group. The cardiac NE levels in RSP+YHB+LPS and RSP+LPS groups were lower compared with YHB+LPS and LPS groups, respectively. However, there was no marked difference in plasma NE concentration between RSP+YHB+LPS and RSP+LPS groups (*Figure 4C and D* *)*. These results indicated that RSP abolished YHB-induced an increase in NE level in the heart, but not in plasma, in LPS-challenged mice. Furthermore, LPS markedly decreased the left ventricular EF and increased myocardial and circulatory TNF-α and NO contents as well as myocardial iNOS expression, all of which were inhibited by YHB pretreatment. RSP not only partly reversed the inhibitory effects of YHB on cardiac TNF-α and NO production as well as iNOS expression, but also abolished the protection of YHB against cardiac dysfunction in LPS-challenged mice. In contrast, RSP did not significantly reverse the inhibitory effects of YHB on plasma TNF-α and NO production in LPS-treated mice (*Figure 4E–J*).

### Both Reserpine and Yohimbine Suppressed Cardiomyocyte Apoptosis in Lipopolysaccharide-challenged Mice

**Figure 5:**
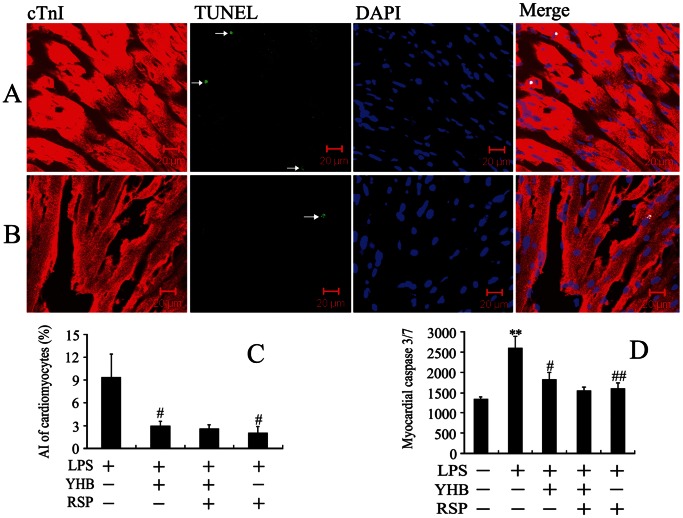
Effects of YHB or/and reserpine (RSP) on the cardiomyocyte apoptosis in LPS-challenged mice. (A and B) Representative confocal images of cardiac troponin I, DAPI and TUNEL-stained cardiac sections are shown from LPS and YHB+LPS groups, respectively. (C) Apoptotic index (AI) of cardiomyocytes at 12 h after LPS injection (*n* = 10). (D) Cardiac caspase 3/7 activity at 2 h after LPS injection (*n* = 10). **P<0.01 compared with control group; ^#^ *P*<0.05, ^##^ *P*<0.01 compared with LPS group.

As shown in *Figure 5A and B*, more apoptotic cardiomyocytes were observed at 12 h after LPS treatment in the LPS group than YHB+LPS group. Compared with control, LPS significantly increased cardiac caspase 3/7 activity and cardiomyocyte apoptosis, both of which were partly inhibited by YHB or RSP pretreatment. Moreover, RSP did not eliminate the inhibitory effects of YHB on LPS-induced cardiac caspase 3/7 activation and cardiomyocyte apoptosis (*Figure 5C and D*).

### β_1_-AR Antagonist Partly Abrogated the Protection of Yohimbine against Lipopolysaccharide-induced Cardiac Dysfunction

**Figure 6:**
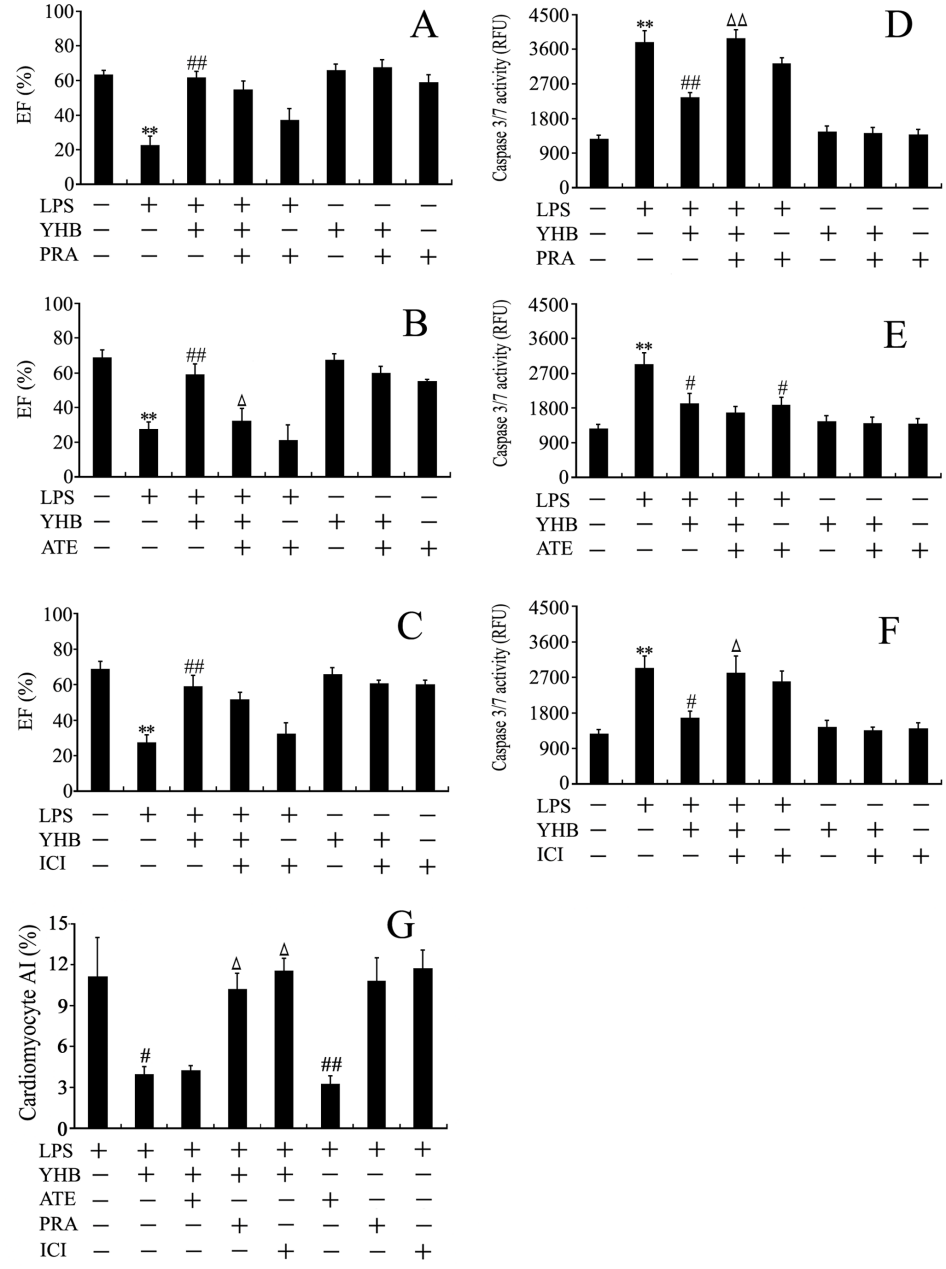
Effects of prazosin (PRA), atenolol (ATE) and ICI 118551 (ICI) on the cardioprotective action of YHB in LPS-challenged mice. PRA (α_1_-AR antagonist, 2 mg/kg), ATE (β_1_-AR antagonist, 10 mg/kg), ICI (β_2_-AR antagonist, 10 mg/kg) or vehicle were delivered intraperitoneally and followed by intragastrical administration of YHB (1 mg/kg) or water. LPS (20 mg/kg) or normal saline was injected intraperitoneally 1 h after treatment with YHB or water. (A, B and C) The left ventricle EF was examined at 12 h after LPS injection (*n* = 8). (D, E, F) Cardiac caspase-3/7 activity (*n* = 8) and (G) cardiomyocyte apoptotic index (AI, *n* = 10) were detected at 2 h and 12 h after LPS injection, respectively. **P<0.01 compared with control group; ^#^ *P*<0.05, ^##^ *P*<0.01 compared with LPS group; ^Δ^ *P*<0.05, ^ΔΔ^ *P*<0.01 compared with YHB+LPS group.

In order to identify which subtype of AR mediates the protection of YHB - enhanced cardiac NE against LPS-induced cardiac dysfunction, we further investigated the effects of prazosin (PRA, α_1_-AR antagonist), atenolol (ATE, β_1_-AR antagonist ) and ICI 118551 (ICI, β_2_-AR antagonist) on the protection of YHB against cardiac dysfunction in LPS-challenged mice. As shown in *Figure 6A–C*, only blockage of the β_1_-AR significantly reduced the protective effect of YHB on LPS-induced cardiac dysfunction. The left ventricular EF in ATE+YHB+LPS group was lower than that in YHB+LPS group. There was no marked difference in left ventricular EF between PRA+YHB+LPS or ICI+YHB+LPS group and YHB+LPS group. PRA, ATE and ICI had no significant effect on LPS-decreased EF.

Prazosin and ICI 118551, but not atenolol, diminished anti-apoptotic action of yohimbine in the myocardium of lipopolysaccharide-challenged mice, and atenolol inhibited LPS-induced cardiomyocyte apoptosis.

As shown in *Figure 6D–G* *,* LPS activated cardiac caspase 3/7 and increased TUNEL-positive cardiomyocytes, both of which were suppressed by YHB pretreatment. PRA, an α_1_-AR antagonist and ICI, a β_2_-AR antagonist, but not ATE, a β_1_-AR antagonist, abolished anti-apoptotic action of YHB in LPS-challenged mice. Moreover, there was no distinct difference in cardiac caspase 3/7 activity and cardiomyocyte AI between LPS group and PRA+LPS or ICI+LPS groups. In contrast, Blockage of β_1_-AR with ATE partly decreased LPS-caused cardiac caspase 3/7 activation and cardiomyocyte apoptosis.

## Discussion

The present study demonstrated that YHB significantly reversed the LPS-induced decreases in left ventricular EF, SV and CO as well as increase in LVESV in mice. It is well known that the markedly reduced LV preload after LPS challenge does not allow the interpretation that the decreased EF reflect reduced myocardial contractility. We further observed that LPS or/and YHB did not markedly affect LV preload, as indicated by the lack of significant variation in LVEDV of mice exposed to YHB and LPS at 12 h after LPS injection. Thus, these results suggest that YHB can attenuate cardiac contractile dysfunction during LPS-induced sepsis. We also demonstrated that YHB prevented hepatic injury, but not kidney and lung injuries in LPS-challenged mice. The previous study showed that α_2A_-AR antagonist, BRL-44408 maleate, suppressed sepsis-induced liver injury via blocking α_2A_-AR on hepatic Kupffer cells [10]. These results suggest that YHB improves LPS-induced cardiac dysfunction maybe via directly acting on the heart.

Increasing evidence has demonstrated that TNF-α and NO are important factors contributing to myocardial dysfunction during sepsis [5], [18]. Inhibition of TNF-α and inducible nitric oxide synthase (iNOS) expression improve LPS-induced cardiac function [19], [20]. Moreover, LPS significantly increased cardiac TNF-α production at 1–4 h, while a robust increase in cardiac NO production was found at 12 h after LPS administration [18]. Therefore, we decided to first focus on TNF-α and NO production in LPS-treated mice and examined the effects of YHB on cardiac and plasma TNF-α levels at 1 h and NO concentration at 12 h as well as myocardial iNOS expression at 6 h after LPS treatment. The results showed that YHB suppressed LPS-induced iNOS expression in the myocardium, TNF-α and NO production in the heart and plasma. It has demonstrated that monocytes and macrophages, especially Kupffer cells, are the predominant source of circulating TNF-α in response to LPS [10], [21]. Several studies also showed that LPS increased systemic NE levels [22], and increased NE potentiated LPS-induced TNF-α production through an α_2_-AR-dependent pathway in macrophages [23] and Kupffer cells [10]. Thus, YHB might attenuate plasma TNF-α level in LPS-challenged mice via blocking α_2_-AR in macrophages. On the other hand, cardiac infiltrated macrophages mediate LPS-induced myocardial contractile dysfunction via TNF-α and NO [13]. Accordingly, YHB may reduce cardiac TNF-α and NO production in LPS-challenged mice through antagonizing α_2_-AR in infiltrated macrophages. However, cardiomyocytes themselves are the important local source of TNF-α and NO during endotoxemia [5], [24]. Although α_2_-AR is not found in cardiomyocytes, α_2_-AR are presented in cardiac sympathetic nerve presynaptic membrane, vascular endothelial cells and smooth muscle cells. Therefore, we don’t exclude one possibility that YHB decreases cardiac iNOS expression, TNF-α and NO production in LPS-challenged mice via a macrophage-independent manner. To test this possibility, we examined the cardiac distribution of α_2A_, α_2B_ and α_2C_-AR as well as effects of YHB on α_2A_, α_2B_ and α_2C_-AR levels in the heart and lung of mice after LPS challenge. The results showed that LPS treatment for 4 h markedly decreased cardiac α_2B_-AR, but not α_2A_ and α_2C_-AR levels in mice, and YHB administration significantly down-regulated the expression of cardiac α_2A_, α_2B_ and α_2C_-AR in the presence of LPS compared with control. Some studies have demonstrated that selective intracoronary blockade of α_2_-AR improved subendocardial blood flow and regional contractile function during coronary hypoperfusion [25], [26]. These data indicate that protection of YHB against LPS-induced cardiac dysfunction might be partly due to the inhibition of cardiac α_2_-AR expression. This needs to be further investigated. In particular, the current study showed that LPS and YHB did not affect the protein levels of α_2_-AR subtypes in the lung, whereas YHB down-regulated cardiac α_2A_-AR protein level in LPS-challenged mice at 4 h after LPS injection. Moreover, immunofluorescence analysis demonstrated that α_2A_-AR was mainly distributed in cardiac sympathetic nerve presynaptic membrane. Although the mechanisms for decreased level of cardiac α_2A_-AR by YHB in LPS-challenged mice is still unclear, these findings indicate that cardiac presynaptic α_2A_-AR may be involved in the protection of YHB against LPS-induced cardiac dysfunction. It was reported that endogenous release of NE activated cardiac sympathetic nerve presynaptic α_2_-AR and in turn led to an inhibition of further release of this neurotransmitter, and blockade of presynaptic α_2_-AR with YHB augmented sympathetic field stimulation-induced NE release [15]. Thus, we detected cardiac NE release in YHB or/and LPS-treated mice. The results demonstrated that LPS challenge for 2 h significantly elevated plasma NE level, but not cardiac NE content. This difference is likely due to cardiac endogenous NE that inhibits its further release via activating presynaptic α_2A_-AR during endotoxemia. In contrast, YHB significantly increased cardiac NE concentration in LPS-challenged mice. In order to identify the role of YHB-promoted cardiac release of NE in the protection of YHB against LPS-induced cardiac dysfunction, we exhausted cardiac NE by injection of reserpine as previously described [17]. The results showed that reserpine significantly reduced cardiac NE content, but not plasma NE level on the 4th day after last reserpine administration in LPS or YHB and LPS -challenged mice. Besides sympathetic nerve endings, plasma NE is also derived from adrenal medulla, intestine and macrophages during sepsis [27], [28]. Koganei, et al. observed that YHB enhanced adrenal NE release in response to splanchnic nerve stimulation [29], and Martínez-Olivares, et al. found that recovery of NE concentrations in the adrenal gland occurred on the fourth day after reserpine treatment [17], suggesting that YHB may increase adrenal NE release on the fourth day after reserpine treatment in endotoxemic mice. In addition, Flierl, et al. demonstrated that exposure of phagocytes from the mice 4 days after reserpine treatment to LPS led to a release of catecholamines, and macrophages as well as neutrophils were a new source of catecholamines during LPS stimulation [28]. These investigations may explain why reserpine only exhausts cardiac NE, but not decreases the circulating NE level, in LPS or YHB and LPS-treated mice. Furthermore, we observed that YHB increased plasma NE concentration, inhibited plasma TNF-α and NO levels in LPS-challenged mice, which were not abrogated by reserpine treatment. However, YHB elevated cardiac NE content, suppressed cardiac iNOS expression, TNF-α and NO production and improved cardiac dysfunction in LPS-challenged mice, all of which were reversed by reserpine pretreatment. These findings indicate that YHB inhibits cardiac iNOS expression, TNF-α and NO production and improves cardiac dysfunction in LPS-challenged mice, at least in part, through blocking presynaptic α_2A_-AR and in turn promoting cardiac NE release. However, the mechanisms responsible for inhibition of LPS-induced cardiac iNOS expression, TNF-α and NO production by endogenously increased NE in the presence of YHB remain to be further investigated.

It has demonstrated that cardiomyocyte apoptosis plays an important role in LPS-induced cardiac dysfunction [4], [6]. Herein, we further investigated the effect of YHB on LPS-induced cardiomyocyte apoptosis. The results showed that YHB inhibited LPS-induced cardiac caspase-3/7 activation and cardiomyocyte apoptosis. Some researchers found that caspase-3 inhibition ameliorated sepsis-induced decreased cardiomyocyte contractility and blocked the blunted contractile response of NE [4], [30]. Thus, inhibition of caspase-3 may be partly responsible for the beneficial effect of YHB on LPS-induced cardiac dysfunction. However, the inhibitory effects of YHB on LPS-induced cardiomyocyte apoptosis were not reversed by reserpine. To our surprise, reserpine reduced cardiac NE content, but not plasma NE concentration, and significantly suppressed cardiomyocyte apoptosis in LPS-challenged mice. These findings indicate that cardiac NE is an important inducer of cardiomyocyte apoptosis during endotoxemia. To our knowledge, this is first report to demonstrate that cardiac NE plays a vital role in LPS-induced cardiomyocyte apoptosis. Because α- and β-AR signal pathways differentially regulate cardiomyocyte apoptosis [31], the above results cannot establish whether increased cardiac NE is involved in the inhibitory effects of YHB on LPS-induced cardiomyocyte apoptosis. Therefore, we further examined the effects of α_1_-, β_1_- and β_2_-AR antagonists on the inhibitory effects of YHB on LPS-induced cardiomyocyte apoptosis. The results showed that α_1_-AR antagonist or β_2_-AR antagonist, did not inhibit LPS-induced cardiomyocyte apoptosis, but abolished the inhibitory effects of YHB on LPS-induced cardiomyocyte apoptosis. These data indicate that activation of α_1_-AR as well as β_2_-AR by increased cardiac NE partly contributes to inhibitory effects of YHB on LPS-induced cardiomyocyte apoptosis. In contrast, we also found that β_1_-AR antagonist inhibited LPS-induced cardiomyocyte apoptosis, suggesting that β_1_-AR activation participates in LPS-induced cardiomyocyte apoptosis. As mentioned above, our results showed that NE and resulting β_1_-AR activation were involved in LPS-induced cardiomyocyte apoptosis, whereas the presence of YHB helped to suppress the harmful effects of NE and β_1_-AR activation on cardiomyocyte apoptosis in the endotoxemic mice. The reason for this phenomenon may be related to the inhibition of myocardial TNF-α production as well as activation of α_1_-, and β_2_-AR by elevated cardiac NE in the presence of YHB in endotoxemic mice. We have demonstrated that NE at less than 20 nM did not induce adult rat cardiomyocyte apoptosis, but promoted LPS or TNF-α -induced cardiomyocyte apoptosis (data not shown). Therefore, YHB pretreatment increases cardiac NE levels, in turn inhibits myocardial TNF-α production and stimulates α_1_-AR as well as β_2_-AR, which maybe abrogates enhancement of cardiomyocyte apoptosis by NE and resulting β_1_-AR activation in the endotoxemic mice.

It is well known that NE has a direct contractile effect on cardiomyocytes. We also investigated the effects of α_1_-, β_1_- and β_2_-AR antagonists on the inhibitory effects of YHB on LPS-induced cardiac dysfunction. The results demonstrated that only blockade of β_1_-AR partly abolished the inhibitory effect of YHB on LPS-induced a decrease in EF, suggesting that YHB improved LPS-induced cardiac contractile dysfunction partly via increasing cardiac release of NE and thus activating β_1_-AR.

However, there are limitations in the present study. First, the assessment of myocardial function is restricted to echocardiographic measurement and the use of mice makes hemodynamic measurements technically difficult. Second, the endotoxemic mouse model is an accelerated model of sepsis, in which LPS triggers an inflammatory cascade and causes a rapid decline in cardiac function. This kind of model does not fully mimic cardiovascular dysfunction in septic patients. Thus, the rat model of cecal ligation and puncture is needed to further determine the clinical significance of the findings in this study.

**Figure 7:**
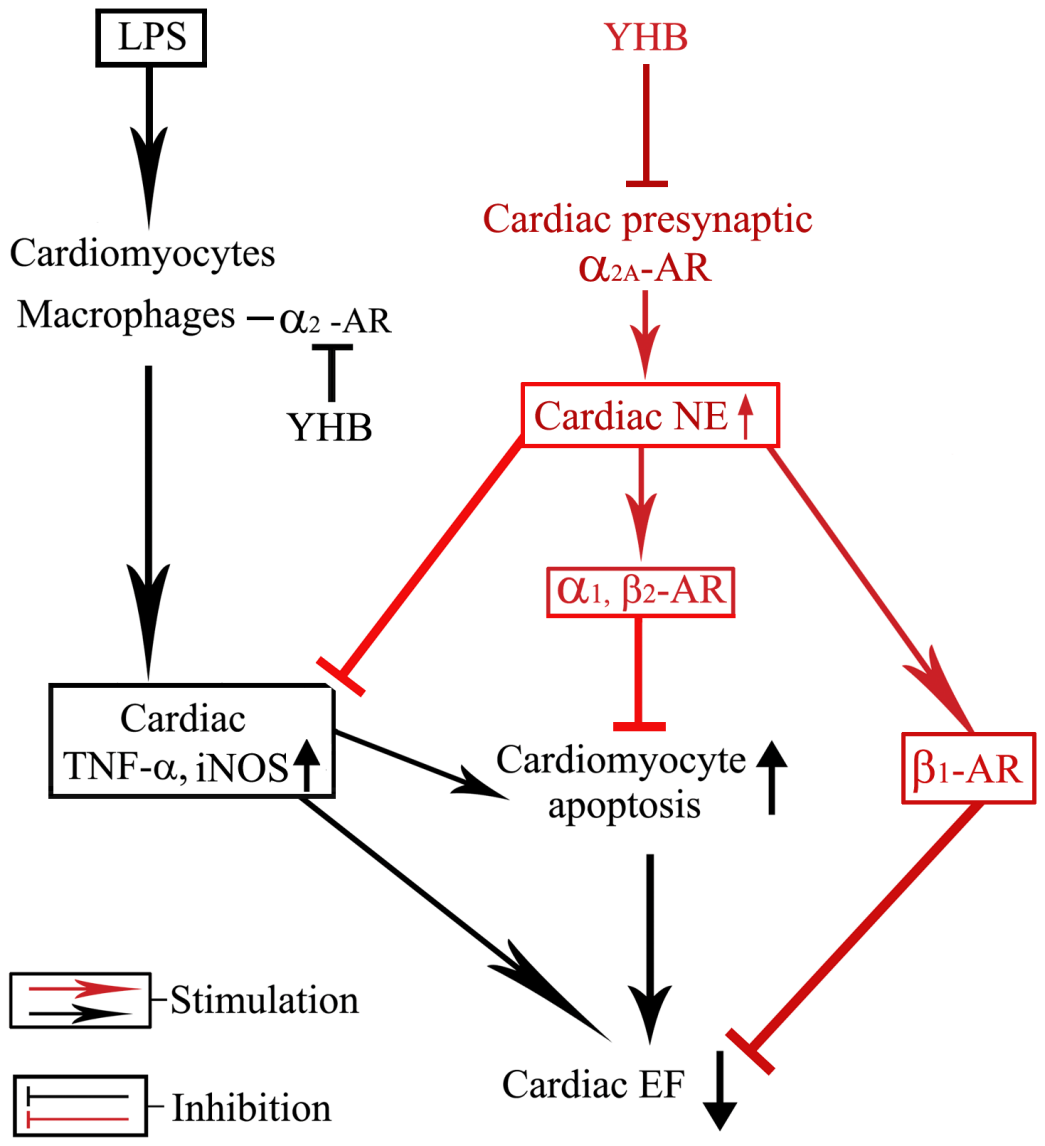
Proposed mechanisms involved in improvement of LPS-induced cardiac dysfunction by YHB. Besides α_2_ -AR in infiltrated macrophages, YHB blocked cardiac presynaptic α_2A_-AR and in turn increases cardiac NE release during endotoxemia. Elevated cardiac NE inhibits cardiac TNF-α and iNOS expression, attenuates cardiomyocyte apoptosis via stimulating α_1_-AR and β_2_-AR, directly activates β_1_-AR and thereby improves LPS-induced decreased EF.

Taken together, the central finding of the present study is that YHB improves cardiac dysfunction in LPS-challenged mice, at least in part, through blocking cardiac presynaptic α_2A_-AR and in turn increasing cardiac NE release. Elevated cardiac NE in the presence of YHB improves cardiac contractile function via inhibiting cardiac iNOS expression and TNF-α production, directly activating β_1_-AR and suppressing cardiomyocyte apoptosis through α_1_- and β_2_-AR in LPS-challenged mice (*Figure 7*). These findings have demonstrated a novel pathway by which YHB improves cardiac dysfunction in LPS-challenged mice, and suggest that inhibition of α_2A_-AR may provide a novel therapeutic strategy for myocardial dysfunction in septic patients.
